# Human Polyomaviruses and Papillomaviruses

**DOI:** 10.3390/ijms19082360

**Published:** 2018-08-10

**Authors:** Ugo Moens

**Affiliations:** Molecular Inflammation Research Group, Department of Medical Biology, Faculty of Health Sciences, University of Tromsø, The Artic University of Norway, NO-9037 Tromsø, Norway; ugo.moens@uit.no; Tel.: +47-7764-4622

Human polyomaviruses (HPyV) and papillomaviruses (HPV) were originally grouped in the family *Papovaviridae* because of their similarity in morphology and genome organization but are now classified in the separate families of *Polyomaviridae* and *Papillomaviridae*, respectively [1,2]. Members of both families play a causative role in human cancers. Merkel cell polyomavirus is implicated in Merkel cell carcinoma, an aggressive skin cancer, and the human polyomavirus species BK (BKPyV) and JC (JCPyV) have been associated with prostate and colorectal cancer, respectively [3,4]. The recent identification of novel human polyomaviruses has invigorated the investigation of a possible role for these viruses in malignancies and other diseases. The oncogenic properties of HPyV depend on the action of the viral proteins large T-antigen and small t-antigen. High-risk HPV (HR-HPV) are members of the *Papillomaviridae* that can cause human cancer mainly through the action of their viral oncoproteins E6 and E7. Epidemiological studies point to an etiological role of HR-HPV predominantly in cervical cancer, but also penile and anal cancers, and head and neck squamous carcinomas [5,6]. Therefore, HPV vaccination should not be restricted to women, but also include men at risk of developing HPV-induced cancers [7].

In the Special Issue “Human Polyomaviruses and Papillomaviruses”, interesting findings on the role of the BKPyV agnoprotein in viral release, the biological importance of mutations in the promoter of HPyV9 variants, the epidemiology of HR-HPV in cervical and oropharyngeal cancer, the role of HPV in autophagy, and mechanisms of transformation are presented.

So far, 14 HPyV have been isolated from different human samples [1]. BKPyV and JCPyV were the first HPyV to be identified almost 50 years ago. JCPyV is associated with progressive multifocal leukoencephalopathy, whereas BKPyV is linked with polyomavirus-associated nephropathy and hemorrhagic cystitis in kidney and bone marrow transplant patients [8], respectively. As mentioned above, a probable role in cancer for these two viruses has also been suggested. The mechanism of HPyV shedding from infected cells remains poorly understood. BKPyV and JCPyV are the only HPyV that encode agnoprotein, an approximately 70-amino acid-long polypeptide that is expressed during the late phase of infection and that sustains viral infectivity [9]. The study by Panou et al. demonstrates that BKPyV agnoprotein is essential for the nuclear egress of virions, and this requires the interaction with the cellular protein α-soluble *N*-ethylmaleimide-sensitive fusion attachment protein (α-SNAP) [10]. This finding indicates that agnoprotein and α-SNAP may be druggable targets to prevent BKPyV shedding from infected cells. Another intriguing feature of HPyV biology is the genomic diversity of different HPyV species. The genomes of HPyV species show considerable sequence identity in their gene-encoding region, but little or no homology in the promoter/enhancer region [11]. In addition, clinical isolates of the same HPyV species display mutations and rearrangements in this region. The biological importance of these changes for the viral life cycle and the pathological properties of HPyV are incompletely understood. Moens and co-workers compared the transcriptional activity of two HPyV9 variants [12]. They used the HPyV9 strain that was originally detected in the serum of a renal transplant patient and a variant (UF-1) isolated from the peripheral blood monocytes of an AIDS patient. The UF-1 strain has three additional putative binding sites for transcription factor Sp1. The basal promoter activity of the UF-1 was stronger than that of the original HPyV9 strain in seven different cell lines and was more potently induced by large T-antigen in most of the cell lines examined. The Sp1 sites were required for large T-antigen activation of the UF-1 promoter activity [12]. This study confirms that rearrangements in the promoter region of the virus may affect the biological features of the virus.

The role of HPV in cervical cancer is well established, and there is increasing epidemiological evidence that HPV contributes to other malignancies. Four papers deal with different aspects of the molecular mechanisms by which HPV induces tumorigenesis [13,14,15,16]. Yeo-Teh and colleagues provide an update of the molecular mechanisms of HR-HPV-induced cervical cancer. Their review focuses on the interference of the oncoproteins E6 and E7 with hallmarks of cancer, including proliferation, immortalization, evasion of apoptosis, immune responses, and DNA damage. Therapeutics against HPV, including prophylactic vaccination and other treatments in clinical trials, e.g., a CRISPR/Cas9-based strategy and drugs against E6 and E7, are discussed [13]. Nilsson et al. reviewed the molecular mechanisms by which HPV employs cellular DNA damage response (DDR) factors such as BRCA1, BCLAF1, BARD1, and TRAP150 to assist in the replication of its genome and to recruit splicing factors (e.g., SF3b and U2AF65) and other RNA processing factors (e.g., hnRNP C, HuR) to induce HPV late gene expression [14]. HPV infections also alter the alternative splicing of cellular mRNAs, including transcripts of DDR factors and RNA-processing proteins. Once again, the hijacking of the DDR and splicing factors by HPV illustrates how these viruses restrict their genome size by usurping cellular factors rather than by encoding their own proteins and underscores the ingenuity of HPV to exploit the cellular machinery for their benefit to ascertain a successful life cycle. The infection of cervical epithelial cells is associated with abnormal growth referred to as cervical intraepithelial neoplasia (CIN). Barillari et al. evaluated the role of HPV oncoproteins, matrix metalloproteinases (MMPs), and HIV protease inhibitors in the development of CIN [15]. HR-HPV E5, E6, and E7 can stimulate the expression of MMP-2 and MMP-9, and this is associated with the development and progression of CIN. However, anti-MMP drugs have shown little therapeutic value. Women co-infected by both HR-HPV and HIV have a higher incidence of CIN compared to their HIV-negative counterparts. HIV protease inhibitors reduce the growth and viability of HR-HPV-transformed epithelial cells and cause tumor regression in animal models. Moreover, preclinical and clinical studies showed that HIV-protease inhibitors caused the regression or the complete remission of high-grade CIN also in HIV-negative women [15]. However, additional studies are required to develop safer and more effective drugs. HPV DNA integration is an early step in cervical carcinogenesis, and the integration sites are unique and highly specific for a patient’s tumor [17]. Autophagy is a cellular process that degrades and recycles proteins and organelles in lysosomal vacuoles [18]. Autophagy is deregulated in various human pathologies, including cancer, but also viral infections may interfere with the autophagic pathways [19]. Mattoscio et al. reviewed the molecular mechanism by which HPV16 interferes with autophagy to promote its life cycle in infected host cells. Additionally, the authors discuss how HPV16 exploits the modulation of the autophagy processes to promote cancer progression and describe similarities and differences to other oncogenic viruses such as EBV, human herpes virus-8, human T-cell leukemia virus type 1, hepatitis B virus, and hepatitis C virus [16]. Drugs that prevent HPV16 to interfere with autophagy may offer potential therapeutic treatments.

In this Special Issue, three epidemiological studies address the association of HPV with risk co-factors, namely, smoking, alcohol, and co-infection with other viruses [20,21,22]. A study with 40 esophageal squamous cell carcinoma specimens from patients from Malawi demonstrates that six samples (15%) were positive for HPV16 [20]. One of three patients with dysplastic epithelium of the esophagus was also HPV16-positive, while none of the normal epithelium samples (*n* = 12) contained HPV16 DNA. However, the genome copy number per tumor cell was low (0.001–2.5 copies/tumor cell). HPV genotypes 18, 31, 45, 52, and 58 were not found. Although the International Agency of Research of Cancer (IARC) declared that there is sufficient evidence to associate HPV 16 with oral cancers [23], Gessner et al. found that the differences in HPV prevalence between tumor patients and non-tumor patients were not statistically significant, jeopardizing a role for HPV in esophageal cancer. The authors propose that a “hit-and-run” mechanism cannot be excluded. Their work also demonstrates that HPV positivity among esophageal squamous cell carcinoma patients was not significantly associated with smoking and alcohol consumption, but a relatively small cohort was examined [20]. The effect of smoking on the expression of E7 in cervicovaginal samples from 1473 women was investigated [22]. Of these women, 53% were non-smokers, 41% were smokers, and 6% were ex-smokers. It was found that the odds of being HR-HPV-positive were almost twofold higher for smokers than for non-smokers, whereas there was no statistically significant difference for non-smokers compared to ex-smokers. Despite an increased tendency for E7 positivity in smokers compared to non-smoking women, the difference was not statistically significant. The discrepancy between the results of HR-HPV DNA and E7 protein incidence may be explained by the different sensitivity of the methods. Detection of HR-HPV DNA was based on a sensitive PCR, whereas a less sensitive sandwich ELISA assay was used to detect the E7 protein. Co-infection with other oncogenic viruses is considered a contributing factor in cervical cancer [24]. Drop and colleagues examined the co-presence of HPV (28 different genotypes), BKPyV, and Epstein-Barr virus (EBV) DNA in oral, oropharyngeal, and laryngeal squamous cell carcinomas in a Polish cohort (*n* = 146) [21]. The patient group consisted of 128 (88%) men with a history of smoking (71%) and alcohol abuse (60%). Eighty-one (55%) of the samples were positive for HPV DNA, but the genotypes were not mentioned. Co-infection was detected in 82 (56%) patients, HPV/EBV double infection being the most common (*n* = 28; 34%). Although this study shows that co-infection with potential oncogenic viruses may play a role in the initiation and progression of these cancers, larger populations and the correlation with smoking and alcohol abuse must be further explored. Moreover, chronic inflammation with infiltrating EBV-positive blood cells should be considered when examining tumor tissues for the presence of EBV DNA.

Two studies aimed at identifying possible biomarkers for HR-HPV-positive cancers. The group of Ramqvist compared the expression of cancer- and immune-related proteins in HPV-positive, HPV-negative tonsillar, and base of tongue squamous cell carcinomas and normal tissues [25]. The expression of surface immunoregulatory proteins, chemokines, and cytokines differed significantly between HPV-positive, HPV-negative tumors, and normal tissues and could be used to identify therapeutic targets, to determine the tumor stage, and to predict the clinical outcome. Carow et al. showed that the viral-cellular junction sequences are specific for each tumor, and most tumors showed intra-tumor homogeneity with respect to junction distribution [26]. The authors also analyzed the sera from 21 patients for the presence of cell-free junction fragments. Such sequences could be amplified from the sera of five of the patients. The authors found a higher, although non-significant, detection rate of junction fragments in the sera of relapsed patients than in those of patients with primary tumors. Among the patients with primary tumors, the detection rate of junction DNA correlated significantly with a reduced recurrence-free survival. Hence, HR-HPV-cellular junction sequences can be used as molecular markers for assessing intra-tumor heterogeneity, for the detection of circulating tumor DNA in sera, and for prognosis [26].

This Special Issue offers an interesting perspective on the epidemiology of HR-HPV in different cancers, the mechanisms by which these viruses target host cell proteins to replicate their genome and to induce cancer, the role of co-factors such as smoking and co-infection, and possible novel therapeutic strategies. As for HPyV, this Special Issue provides novel information on the nuclear egress mechanism of BKPyV and opens the possibility of developing strategies to prevent viral release from infected cells. Moreover, studies with HPyV9 variants support previous observations that rearranged transcription control regions affect the biological properties of the virus, as previously shown for BKPyV and JCPyV.
